# Antithrombin III prevents progression of chronic kidney disease following experimental ischaemic‐reperfusion injury

**DOI:** 10.1111/jcmm.13261

**Published:** 2017-08-02

**Authors:** Jianyong Yin, Feng Wang, Yiwei Kong, Rui Wu, Guangyuan Zhang, Niansong Wang, Ling Wang, Zeyuan Lu, Mingyu Liang

**Affiliations:** ^1^ Department of Nephrology Shanghai Jiao Tong University Affiliated Sixth People's Hospital Shanghai China; ^2^ Department of Physiology Medical College of Wisconsin Milwaukee WI USA; ^3^ Center of Systems Molecular Medicine Medical College of Wisconsin Milwaukee WI USA; ^4^ Department of Urology Affiliated Zhongda Hospital of Southeast University Nanjing China; ^5^ Department of Nephrology Renji Hospital Shanghai Jiao Tong University School of Medicine Shanghai China

**Keywords:** acute kidney injury, chronic kidney disease, antithrombin III

## Abstract

Acute kidney disease (AKI) leads to increased risk of progression to chronic kidney disease (CKD). Antithrombin III (ATIII) is a potent anticoagulant with anti‐inflammatory properties, and we previously reported that insufficiencies of ATIII exacerbated renal ischaemia‐reperfusion injury (IRI) in rats. In this study, we examined the characteristic of AKI‐CKD transition in rats with two distinct AKI models. Based on our observation, left IRI plus right nephrectomy (NX‐IRI) was used to determine whether ATIII had therapeutic effects in preventing CKD progression after AKI. It was observed that NX‐IRI resulted in significant functional and histological damage at 5 weeks after NX‐IRI compared with sham rats, which was mitigated by ATIII administration. Besides, we noticed that ATIII administration significantly reduced NX‐IRI‐induced interstitial fibrosis. Consistently, renal expression of collagen‐1, α‐smooth muscle actin and fibronectin were substantial diminished in ATIII‐administered rats compared with un‐treated NX‐IRI rats. Furthermore, the beneficial effects of ATIII were accompanied with decreased M1‐like macrophage recruitment and down‐regulation of M1‐like macrophage‐dependent pro‐inflammatory cytokines such as tumour necrosis factor α, inducible nitric oxide synthase and interleukin‐1β, indicating that ATIII prevented AKI‐CKD transition *via* inhibiting inflammation. Overall, ATIII shows potential as a therapeutic strategy for the prevention of CKD progression after AKI.

## Introduction

Acute kidney injury is a common severe complication characterized by high initial mortality, morbidity and substantial sequelae 1, 2. Due to the great advance in the early diagnosis and renal‐supportive measures, the in‐hospital mortality of AKI has been significantly improved 3, 4, 5. However, the long‐term outcome of AKI survivors remains stagnant. Recent epidemiology studies have demonstrated that patients who recover from AKI have an 8.8‐fold increase in risk for CKD and a 3.3‐fold increase in risk for end‐stage renal disease (ESRD) 6. It is increasingly recognized that AKI is an independent risk factor for CKD 6, 7, and the progression to CKD after the acute recovery phase of AKI is mainly accounted for the poor long‐term prognosis of AKI. Because there are no clinical effective prophylactic interventions to prevent the CKD progression, it is imperative to uncover the underlying mechanisms of AKI‐to‐CKD progression to develop specific and therapeutic strategies to improve the long‐term prognosis of AKI.

Currently, the mechanism of AKI‐CKD transition has not been fully elucidated yet. Various intrinsic pathophysiological processes involved in the progression of CKD after AKI. It is generally thought that maladaptive repair in the tubular, vascular and interstitial compartments is the pivotal factor in the development of interstitial fibrosis 8, 9, 10. Hypoxia 11, fibroblast hyperplasia, immune cells infiltration 12, 13, 14 can both lead to maladaptive repair. Thus, strategies targeting blockade of the aforementioned process will be promising for the prevention of AKI‐CKD transition.

ATIII is not only a powerful anticoagulant in the coagulation cascade but also possesses anti‐inflammatory properties 15, 16. Previous studies have proved that (*i*) administration of exogenous ATIII could protect against renal IRI 17, 18; (*ii*) ATIII‐knockout rats exhibited aggravated renal dysfunction upon renal IRI 19; (*iii*) patients with low activity of ATIII presented a higher risk for developing AKI after cardiac surgery 19. Nonetheless, there is no study to determine the effects of ATIII on the progression of AKI‐to‐CKD so far.

In this study, two different models of AKI were employed to determine whether the severity of AKI would influence the natural course of CKD progression in rats. In addition, exogenous ATIII was administered to the AKI rats to evaluate whether ATIII could prevent renal injury progression following AKI, and the potential mechanisms were also investigated.

## Materials and methods

### Animals and reagents

Male Sprague‐Dawley rats (weighing 200–250 *g*) were purchased from Shanghai Science Academy animal centre. The rats were housed in a pathogen‐free environment and a 12/12‐hrs light/dark cycle. Free access to water and standard rat chow was also provided. After 2 weeks adaption period, the rats were randomly allocated to assigned groups. All animal protocols were approved by the Animal Care and Ethics Committee of Shanghai Jiao Tong University Affiliated Sixth People's Hospital.

### Rat model of AKI and experimental protocols

Study 1 was designed to assess whether the severity of AKI would influence the course of AKI‐CKD transition. Animals were randomly divided into three groups: sham‐operated group (sham, *N* = 6), bilateral IRI group (Bi‐IRI, *N* = 30), left ischaemia‐reperfusion plus right nephrectomy (NX‐IRI, *N* = 30). Briefly, rats were anaesthetized by intraperitoneally injection of sodium pentobarbital (40 mg/kg) and placed on a heating pad (38°C) during surgery. Bi‐IRI (moderate AKI) was induced by clamping the bilateral renal arteries for 40 min with non‐traumatic vascular clamp as previously described. As for the induction of NX‐IRI (severe AKI), rats underwent left kidney ischaemia for 40 min followed by right kidney nephrectomy. Sham‐operated rats were subjected to the same procedures, but only the envelope capsules were removed. The rats were killed at 1, 3 days, 1, 3 and 5 weeks after reperfusion (*N* = 6). Blood, urine and left kidneys were harvested for further analyses.

Study 2 was performed to confirm whether ATIII (Sigma‐Aldrich, St Louis, Mo, USA) administration had beneficial effects on the recovery and preservation of kidney function. Based on the features of two models, NX‐IRI was used in this study. Animals randomly underwent sham‐operation or NX‐IRI procedures. 3 days after surgery, the rats were further randomly divided into following groups: (*i*) sham‐operated treated with intraperitoneally injection of PBS (sham, *n* = 6); (*ii*) NX‐IRI treated with intraperitoneally injection of PBS (NX‐IRI + Veh, *n* = 6); (*iii*) NX‐IRI treated with intraperitoneally injection of ATIII (NX‐IRI + ATIII, *n* = 6). ATIII protein (125μg/kg.W) or vehicle was administered for 32 consecutive days. [Correction added on 27 September 2017, after first online publication: the sentence has been amended for clarity and the value of Antithrombin III was previously incorrect and has been amended in this version.] The ATIII dose used to reduce kidney injury was chosen based on a previous study 20. The effect of ATIII delivery on CKD progression following NX‐IRI was assessed 5 weeks after initial AKI. At the end of the experiment, all the rats were killed, and blood as well as left kidney were harvested for analyses.

### Collection of blood and urine, renal function assessment

At the end‐point of the experiment, all the rats were kept in metabolic cages for 24‐hrs urine collection. Rat blood samples were collected from abdominal aorta and moved into BD Vacutainer^®^ SST™ Serum Separation Tubes (Becton‐Dickinson, Franklin Lakes, NJ, USA). After centrifugation, the serum was then separated and stored at −80°C. Serum and urine creatinine were measured by an automatic biochemical analyser (Hitachi 7600, Tokyo, Japan) to assess the alteration of renal function. Creatinine clearance (Clcr) was calculated by the formula ‘24‐hr creatinine clearance = (urine creatinine concentration × urine flow rate)/serum creatinine concentration and expressed as ml/min.

### Kidney histology, and immunohistochemistry evaluation

Kidney tissues were fixed in 4% paraformaldehyde and embedded in paraffin. Paraffin‐fixed kidney sections were cut into 3 μm sections and stained with haematoxylin and eosin (H&E) for histological assessment. The transverse tubular diameters were calculated as previously described 21. Masson Trichrome and Sirus Red staining were both performed to assess the extent of fibrosis. The fibrotic area was quantified with ImagePro Plus Systems by a pathologist who was blinded to the experimental groups. Rabbit monoclonal anti‐CD86 antibody (Abcam, Cambridge, MA, USA) was used to perform immunohistochemical staining of M1‐like macrophage in renal tissue as described previously 22.

### Western blot analysis

Frozen kidney tissues were homogenized, and protein concentrations were measured by BCA assay (Beyotime, Suzhou, Jiangsu, China). 30 μg total protein samples were separated by immunoblotting as previously described. The blots were then incubated with primary antibodies against collagen‐1, α‐smooth muscle actin (Abcam) and fibronectin (Sigma‐Aldrich), and mouse monoclonal anti‐tubulin antibody (Proteintech, Chicago, IL, USA) was used as loading control. Horseradish peroxidase‐conjugated secondary antibodies (Beyotime) were used, and images were captured by Image Quant LAS 4000 Mini System (GE Healthcare, Pittsburgh, PA, USA). Bands were analysed using Image J software.

### Real‐time PCR

Total RNA from kidney tissues was extracted using Trizol (Invitrogen, Carlsbad, CA, USA). cDNA was generated with random primers using M‐MLV Reverse Transcriptase (Promega, Madison, WI, USA). Real‐time PCR assays were carried out with SYBR Green PCR master Mix (TaKaRa, Dalian, China) using StepOnePlus PCR Systems (Applied Biosystems, Foster City, CA, USA) according to the manufacturer's instructions 23. The specific primers were listed as following; TNFα: 5′‐GTCTGTGCCTCAGCCTCTTC‐3′ (forward) and 5′ ‐TGGAACTGATGAGAGGGAGC‐3′ (reverse); iNOS: 5′‐CTACCTACCTGGGGA ACACCTGGG‐3′ (forward) and 5′‐GGAGGAGCTGATGGAGTAGTAGCGG‐3′ (reverse); rat IL‐1β:5′‐CACCTCTCAAGCAGAGCACAG‐3′ (forward) and 5′‐GGGTTCCATGGTGAAGTCAAC‐3′ (reverse); Data were presented at an amplification number of 2^−ΔΔCT^ normalized to 18S rRNA and compared with sham.

### Statistical analyses

One‐way ANOVA followed by a *post hoc* Bonferroni's test was used to perform multiple comparisons using SPSS software 19.0 (IBM, Armonk, NY, USA). Data were expressed as mean ± standard error (S.E.M.) *P* < 0.05 were considered statistically significant.

## Results

### Comparison of two AKI‐CKD models in rats

**Figure 1:**
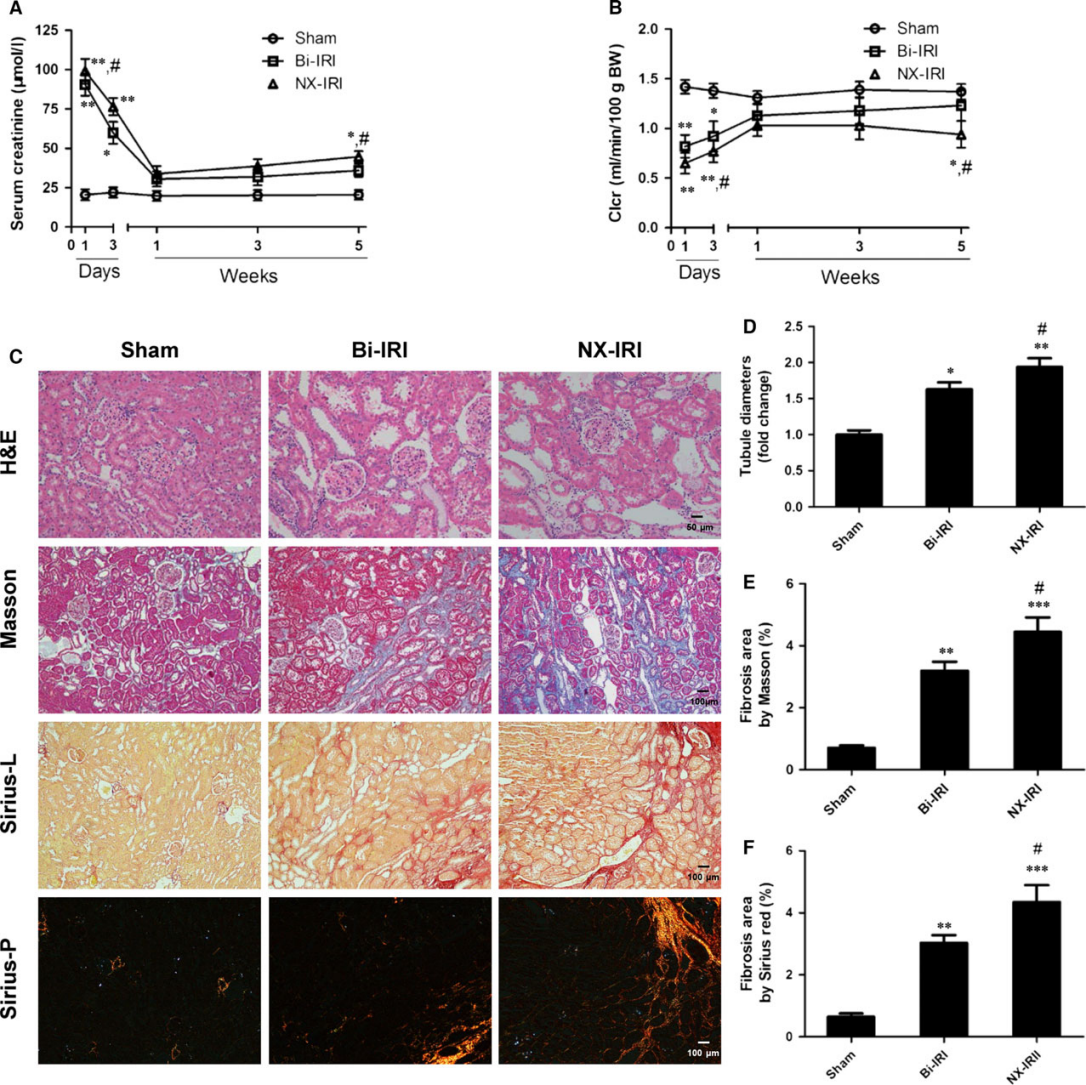
Complete and incomplete recovery of renal function and pathology in two distinct AKI models. Rats were challenged with sham operation, bilateral ischaemia‐reperfusion injury (Bi‐IRI) or left IRI plus with right kidney nephrectomy (NX‐IRI), respectively. Then the rats were killed to obtain blood and kidney tissues at different time‐point as indicated after reperfusion. (**A**) Dynamic changes of serum creatinine over time in AKI rat models, including Bi‐IRI and NX‐IRI. (**B**) Changes in creatinine clearance (Clcr) over time. (**B**) Representative pathological staining including H&E (×200 magnification; Scale bar, 50 μm), Masson's trichrome (×100 magnification; Scale bar, 100 μm) and Sirius red staining in normal and polarized light (×200 magnification; Scale bar, 100 μm) in post‐ischaemic or sham‐operated kidney sections at 5 weeks after reperfusion. (**D**) Relative diameters of transverse tubules at 5 weeks post‐AKI compared with sham. (**E**) Quantitative analysis of renal fibrosis at 5 weeks after reperfusion assessed with Masson staining. (**F**) Quantitative analysis of renal fibrosis at 5 weeks after reperfusion by Sirius red staining. Results are expressed as means ± S.E.M. (*N* = 6 in each group), and statistical significance was determined by one‐way ANOVA followed by Bonferroni's test. *P < 0.05, **P < 0.01, ***P < 0.001 *versus* Sham at same time‐point; ^#^ *P* < 0.05 *versus* Bi‐IRI.

To longitudinally explore the course of CKD progression after AKI and determine whether the severity of AKI was associated with the development of CKD, we used two different AKI models to titrate the severity of AKI. Rats that underwent bilateral ischaemia‐reperfusion injury (Bi‐IRI) or right nephrectomy plus left kidney ischaemia‐reperfusion injury (NX‐IRI) were established and followed for consistent 5 weeks after AKI. Renal functional and morphological alterations were examined over time. As demonstrated in Figure 1A, both Bi‐IRI and NX‐IRI led to a significant increase in serum creatinine (Scr) compared with sham group, and Scr in two AKI groups rapidly declined to approximately normal levels at 1 week after AKI. However, the Scr in NX‐IRI rats at Day 1 was significantly higher than that in Bi‐IRI rats, suggesting that NX‐IRI resulted in more severe injury than Bi‐IRI at the early‐phase of AKI. In addition, the Scr in NX‐IRI rats at 5 weeks was also higher than that in Bi‐IRI and sham rats, while there was no significant difference in Scr between sham and Bi‐IRI. Moreover, there was a similar tendency in the changes of creatinine clearance (Clcr). Clcr in the NX‐IRI group was lower than that in the Bi‐IRI group at Day 3 and did not completely recover to baseline at 5 weeks after AKI (Fig. 1B).

After 5 weeks of reperfusion, kidney sections were stained with haematoxylin–eosin to evaluate the extent of pathological injury. It was observed that Bi‐IRI and NX‐IRI both induced remarkable tubular injury as reflected by loss of brush border and tubular dilation (Fig. 1C, upper panel). Meanwhile, quantitative analysis showed that the median tubular diameter was significantly elevated in comparison with that in Bi‐IRI rats (Fig. 1D). Furthermore, Masson and Sirius red staining of kidney sections showed that NX‐IRI led to a robust collagen deposition at 5 weeks after the initial injury (Fig. 1C). Consistent with renal functional changes, the extent of renal fibrosis was more severe in NX‐IRI rats compared with Bi‐IRI rats as assessed by semiquantitative analysis (Fig. 1E and F). In contrast to the change of Scr, there was modestly but appreciably increased renal fibrosis in Bi‐IRI group compared with sham group. Therefore, we observed the AKI‐to‐CKD progression in two AKI models and found that NX‐IRI induced more severe post‐AKI renal injury, which was characterized by both elevation of Scr and renal fibrosis.

### ATIII administration improved long‐term renal function and attenuated renal fibrosis after NX‐IRI

**Figure 2:**
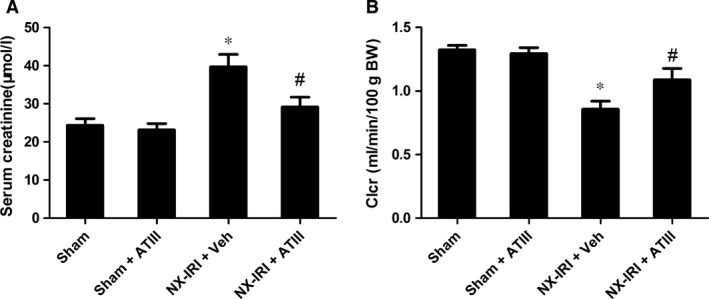
Antithrombin III administration prevented AKI transition to CKD in rats. Rats were randomly allocated with sham operation, left IRI plus with right kidney nephrectomy (NX‐IRI). Rats were intraperitoneally administered Antithrombin III (125μg/kg.W) [Correction added on 27 September 2017, after first online publication: the value of Antithrombin III was previously incorrect and has been amended in this version.] or equal volume of vehicle for 32 consecutive days starting 3 days after NX‐IRI, then were killed to obtain blood and kidney tissues. (**A**) Serum creatinine. (**B**) Creatinine clearance (Clcr). Results are expressed as means ± S.E.M. (*N* = 6 in each group), and statistical significance was determined by one‐way ANOVA followed by Bonferroni's test. *P <* *0.05 *versus* Sham; ^#^ *P *<* *0.05 *versus* NX‐IRI.

**Figure 3:**
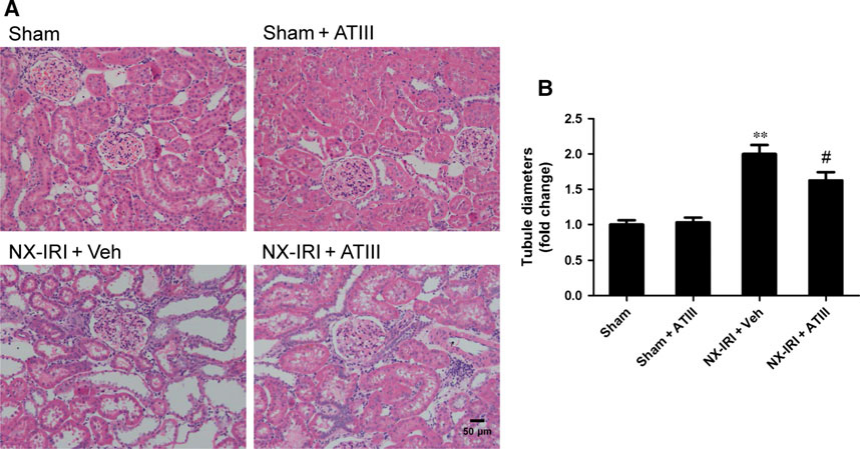
Antithrombin III administration mitigated subsequent renal pathological injury following AKI. Kidney tissues were harvested 5 weeks after left IRI plus with right kidney nephrectomy (NX‐IRI). (**A**) Representative morphology of kidney sections by H&E staining (×200 magnification; Scale bar, 50 μm). (**B**) Quantitative comparison of diameters of tubules. Results are expressed as means ± S.E.M. (*N* = 6 in each group), and statistical significance was determined by one‐way ANOVA followed by Bonferroni's test. **P < 0.01 *versus* Sham; ^#^ *P* < 0.05 *versus* NX‐IRI.

**Figure 4:**
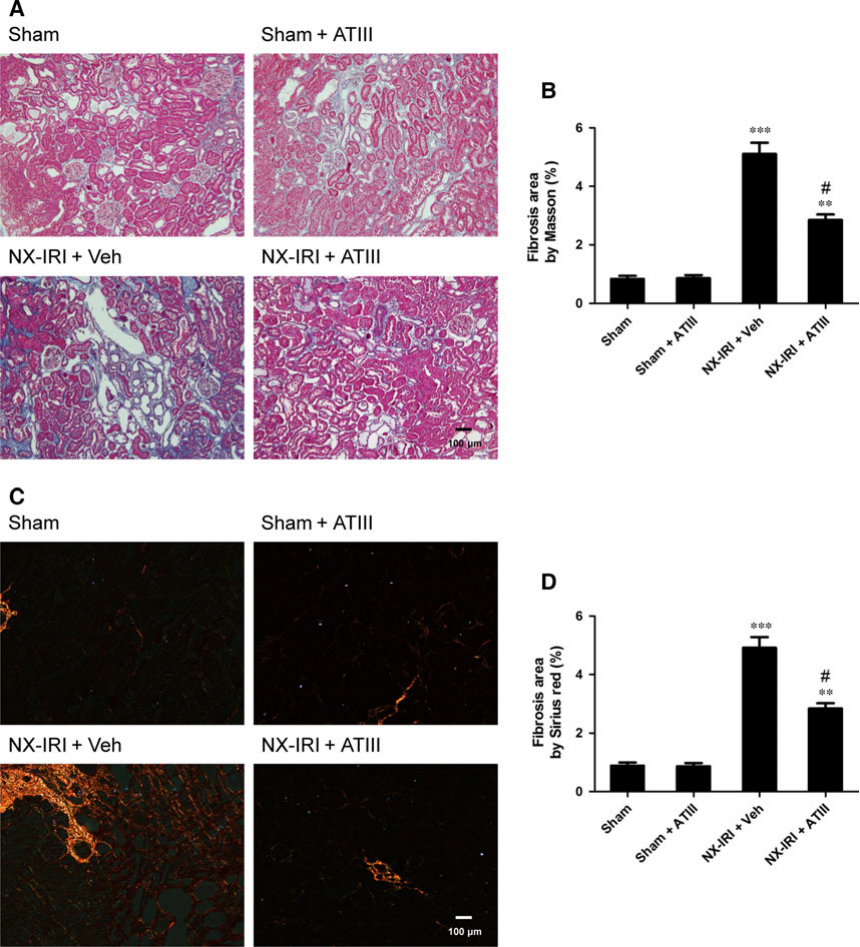
Renal fibrosis was blunted in antithrombin III‐administered NX‐IRI rats. Kidney tissues were harvested 5 weeks after left IRI plus with right kidney nephrectomy. (**A**) Representative photographs of Trichrome‐stained kidney sections (×100 magnification; Scale bar, 100 μm). (**B**) Quantitative assessment of fibrosis by Masson staining. (**C**) Representative photographs of Sirius red‐stained tissues in polarized light. (**D**) Quantitative assessment of fibrosis by Sirius red staining. Results are expressed as means ± S.E.M. (*N* = 6 in each group), and statistical significance was determined by one‐way ANOVA followed by Bonferroni's test. **P < 0.01, ***P < 0.001 *versus* Sham; ^#^ *P* < 0.05 *versus* NX‐IRI.

Because NX‐IRI was a more severe model of AKI and rapidly developed into CKD with both functional and histological impairment, we utilized this model to investigate whether ATIII could inhibit the progression from AKI‐to‐CKD. At 5 weeks after AKI, ATIII administration significantly decreased Scr levels and restored Clcr compared with un‐treated NX‐IRI rats as illustrated in Fig. 2. Consistent with the Scr and Clcr data, histological analysis showed that the tubular enlargement, and brush border loss was ameliorated in ATIII‐administered NX‐IRI rats compared with NX‐IRI + vehicle rats (Fig. 3A). Additionally, tubular diameters in NX‐IRI + ATIII rats were dramatically decreased than that in un‐treated NX‐IRI rats (Fig. 3B). Furthermore, Masson and Sirius red staining collectively manifested that collagen deposition following AKI was dramatically decreased in ATIII‐administered NX‐IRI rats (Fig. 4A and C). To evaluate the effects of ATIII on interstitial fibrosis, quantitative analysis of fibrosis area was also performed and the results revealed that ATIII significantly reduced NX‐IRI‐induced interstitial fibrosis (Fig. 4B and D). Taken together, these results indicated that exogenous ATIII administration prevented the course of AKI‐CKD progression as demonstrated by preserved renal function and ameliorated histological injury.

### ATIII inhibited the expression of pro‐fibrotic markers in rats following NX‐IRI

**Figure 5:**
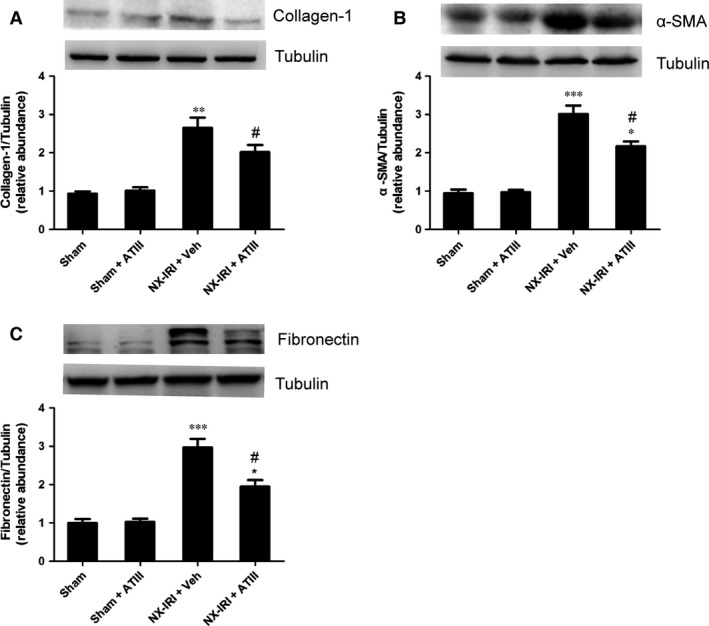
Antithrombin III reduced expression of pro‐fibrotic markers. Kidney tissues were harvested 5 weeks after left IRI plus with right kidney nephrectomy. Immunoblots and semiquantitative analyses of collagen‐1 (**A**), α‐SMA (**B**) and fibronectin (**C**) were performed in different groups. Results are expressed as means ± S.E.M. (*N* = 6 in each group), and statistical significance was determined by one‐way ANOVA followed by Bonferroni's test. *P < 0.05, **P < 0.01, ***P < 0.001 *versus* Sham; ^#^ *P* < 0.05 *versus* NX‐IRI.

To investigate whether pro‐fibrotic molecules were involved in the reno‐protection of ATIII, the expression of related pro‐fibrotic markers were detected. In agreement with fibrosis staining, our data demonstrated that NX‐IRI rats exhibited substantially increased expression of collagen 1, α‐smooth muscle actin (α‐SMA) and fibronectin compared with sham‐operated rats, which was effectively mitigated by ATIII administration (Fig. 5). ATIII might exert its reno‐protective effects *via* inhibiting expression of pro‐fibrotic markers.

### Intrarenal inflammation was blunted in ATIII ‐treated NX‐IRI rats

**Figure 6:**
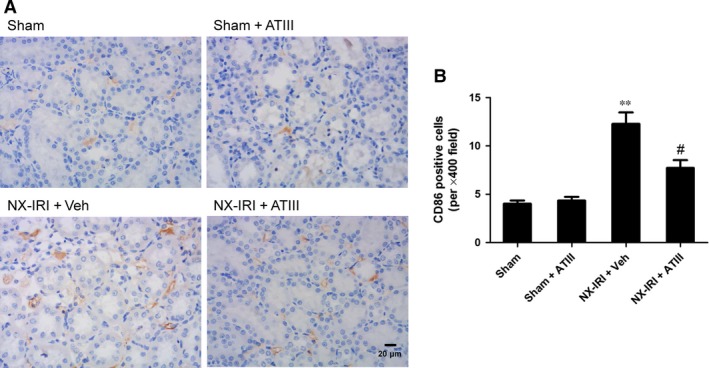
Antithrombin III decreased macrophage infiltration in kidney tissues. Kidney tissues were harvested 5 weeks after left IRI plus with right kidney nephrectomy (NX‐IRI). (**A**) Representative photographs of CD86 immuno‐staining (×400 magnification; Scale bar, 20 μm). (**B**) Quantitative analysis of CD86‐positive cells. Results are expressed as means ± S.E.M. (*N* = 6 in each group), and statistical significance was determined by one‐way ANOVA followed by Bonferroni's test. **P < 0.01 *versus* Sham; ^#^ *P* < 0.05 *versus* NX‐IRI.

**Figure 7:**
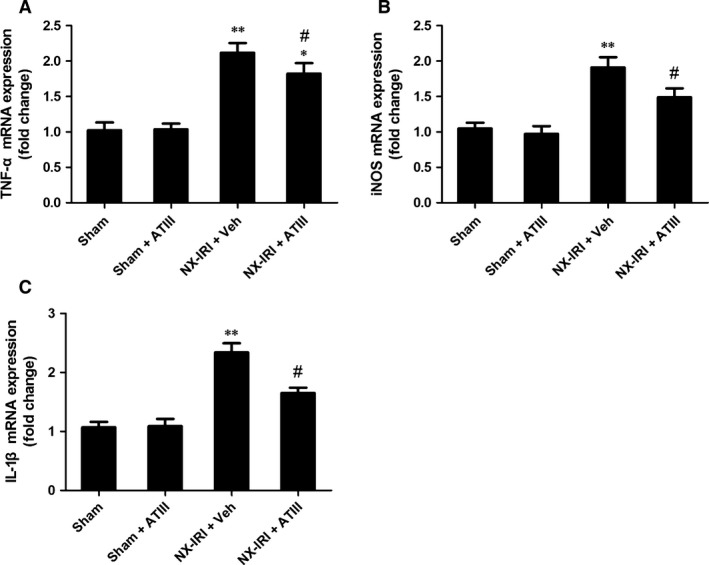
Expression of pro‐inflammatory cytokines in post‐injury kidneys was suppressed after antithrombin III administration. Kidney tissues were harvested 5 weeks after left IRI plus with right kidney nephrectomy (NX‐IRI). (**A**) TNFα mRNA expression. (**B**) iNOS mRNA expression. (**C**) IL‐1β mRNA expression. Results are expressed as means ± S.E.M. (*N* = 6 in each group), and statistical significance was determined by one‐way ANOVA followed by Bonferroni's test.*P < 0.05, **P < 0.01 *versus* Sham; ^#^ *P* < 0.05 *versus* NX‐IRI.

To determine the possible effects of ATIII on renal inflammation during the AKI‐CKD transition, we further examined macrophage infiltration and the expression of pro‐inflammatory cytokines at 5 weeks after initial insult. M1‐like macrophage in kidney sections was stained by using CD86 antibody (Fig. 6). Analysis of immunohistochemistry assay revealed that M1‐like macrophage infiltration was significantly increased 5 weeks after NX‐IRI, while renal M1‐like macrophage infiltration was less in ATIII‐treated NX‐IRI rats than that in vehicle‐treated NX‐IRI rats (Fig. 6). It has been reported that persistent activated macrophage can release pro‐inflammatory cytokines, which may accelerate the development of CKD. To confirm this, we further analysed the alteration in the expression of M1‐dependent pro‐inflammatory cytokines such as tumour necrosis factor α (TNFα), inducible nitric oxide synthase (iNOS) and interleukin‐1β (IL‐1β). The expression levels of aforementioned genes were all massively increased in post‐AKI kidneys and significantly decreased by ATIII administration (Fig. 7). In summary, persistent accumulation of M1‐macrophage was present in kidney following AKI and ATIII may exert its inhibitory effects on AKI‐CKD transition through inhibiting inflammation.

## Discussion

In the current study, we demonstrated that severe initial AKI resulted in accelerated renal functional and pathological damage in rats in comparison with modest AKI. Furthermore, our data proved that exogenous ATIII administration could effectively reduce post‐injury renal damage and delayed the development of fibrosis after AKI. The blockade of AKI‐CKD transition conferred by ATIII might be mediated by inhibition of fibrogenesis and inflammation.

Although mounting evidence has confirmed the close relationship between episodes of AKI and subsequent progression to CKD, a causative link has not been established because of the limitations in clinical studies. It has been accepted that the severity^24^, age^25^, and as well as pre‐existing renal disease, and other co‐morbidities 7, 26, 27 such as diabetes, cardiovascular diseases, are associated with increased risk for CKD progression. These confounding factors make it difficult to interpret the results of clinical study. So it seems that developing more appropriate animal models is very necessary and important to identify the causal correction between AKI and ensuing CKD. The aim of our study was to evaluate whether the severity of AKI had an influence on the development of subsequent CKD by utilizing two different approaches corresponding to the presence *versus* absence of reduced nephron mass. Our data showed that the NX‐IRI (corresponding to severe AKI) led to aggravated renal function and increased fibrosis than BI‐IRI (corresponding to modest AKI) as demonstrated by higher Scr and increased fibrosis area in kidney tissues in NX‐IRI group. Consistently with our findings, previous studies also found that renal functional and histological injury were more severe in NX‐IRI mice 28. Furthermore, clinical studies have showed that the magnitude of acute Scr increase is a useful predictor for the incidence of subsequent CKD 24, 29, which resonated with our data that NX‐IRI rats had higher Scr levels 24 hrs after reperfusion. This phenomenon can be explained by that modest AKI can induce adaptive repair characterized by recovery of renal functional or structural damage, whereas severe AKI resulted in maladaptive repair characterized by scarring and fibrosis of kidney.

Maladaptive repair, including sustained activation of pro‐inflammatory, oxidative stress and pro‐fibrotic pathways is the crucial driving factor for AKI‐CKD transition. Progressive tubulointerstitial fibrosis is the hallmark of AKI‐to‐CKD progression. In agreement with previous studies 28, 30, 31, we observed that both NX‐IRI and BI‐IRI led to increased renal fibrosis following AKI. It was noteworthy that BI‐IRI also caused slightly but significantly increased collagen accumulation at 5 weeks after AKI regardless of no significantly increase in Scr compared with sham, indicating that the histological change of kidney tissues might precede the decline of biochemistry parameters. Moreover, the severity of renal fibrosis represented the progression of AKI‐to‐CKD in some extent. On the one hand, kidney fibrosis staining in AKI rats exhibited increased collagen deposition and up‐regulation of pro‐fibrotic genes than that in sham‐operated rats. On the other hand, ATIII administration restored renal function and attenuated post‐AKI fibrosis, which was accompanied with down‐regulation of related pro‐fibrotic molecules. Taken together, the blockade of progressive fibrogensis might be accounted for the protective effects of ATIII.

However, how ATIII mediated its anti‐fibrosis mechanism following AKI remains unclear. It has been reported that ATIII is a pleiotropic molecules with anti‐coagulation and anti‐inflammation effects. The underlying mechanisms anti‐inflammatory properties of ATIII include inhibiting neutrophils recruitment and leucocytes rolling and infiltration 32, 33, pro‐inflammatory cytokines secretion^34^ and inhibition of nuclear factor (NF)‐κB pathways 35, 36. Previous studies showed that ATIII exerts its reno‐protective effects against renal IRI *via* inhibiting leucocyte activation and oxidative stress. Herein, our data revealed that ATIII administration also had therapeutic effects in preventing chronic renal injury following AKI. Based on previous studies, no evidence revealed that ATIII could directly inhibit pro‐fibrotic pathways. Given the biological properties of ATIII, we suggested that the ATIII‐mediated reno‐protection was mainly attributed to the inhibition of inflammation. This speculation could be supported as follows: (*i*) it has been well‐known persistent inflammation contributed to the development of CKD following AKI, especially the activation of pro‐inflammatory immune cells and excess release of pro‐inflammatory chemokine or cytokines. In agreement with previous studies 13, 37, sustained M1‐like macrophage recruitment was observed in the kidney of NX‐IRI rats, which was significantly inhibited by ATIII administration; (*ii*) up‐regulation of M1‐like macrophage‐dependent cytokines such as TNFα and iNOS was blunted in ATIII‐administered rats; (*iii*) previous study showed that prolonged up‐regulation of IL‐1β contributed the CKD progression after IRI 38, which was consistent with our results; more importantly, ATIII was also able to decrease IRI‐induced up‐regulation of IL‐1β.

There are some limitations of this study. First, there are still no specific markers to distinct macrophage polarization *in vivo*. In the current study, we utilized CD86 to represent M1‐like macrophage, as a relative specific marker for M1‐like macrophage, which was also expressed on monocytes. Thus, the effects of ATIII on macrophage infiltration might be overstated, and ATIII might inhibit renal inflammation *via* promoting the M2 macrophage infiltration. Second, in addition to its anti‐inflammation effects, other mechanisms might involve in its protection against AKI‐CKD transition. It has been reported that ATIII can promote the release of prostacyclin (PGI2) from endothelial cells. PGI_2_ is a powerful vasodilator and may improve the micro‐circulation of kidney and reduce hypoxia, and subsequently inhibited AKI‐CKD transition. Besides, ATIII administration might reduce the oxidative stress levels following AKI. Thus, we could not rule out these mechanisms of ATIII. Finally, our observation duration is only 5 weeks, which may be too short for AKI‐CKD transition. Therefore, further studies will be needed in the future.

In summary, ATIII administration ameliorated both renal dysfunction and fibrosis after AKI in rats. The potential mechanisms were associated with its inhibition on the expression of pro‐fibrotic molecules and M1‐macrophage infiltration. Therefore, ATIII administration may be a promising strategy for prevention of AKI‐to‐CKD progression.

## Conflicts of interests

None.
